# Isolated and Combined Effects of Electroacupuncture and Meditation in Reducing Experimentally Induced Ischemic Pain: A Pilot Study

**DOI:** 10.1155/2011/950795

**Published:** 2010-09-08

**Authors:** Kyung-Eun Choi, Frauke Musial, Nadine Amthor, Thomas Rampp, Felix J. Saha, Andreas Michalsen, Gustav J. Dobos

**Affiliations:** ^1^Chair of Complementary and Integrative Medicine, University of Duisburg-Essen, 45276 Essen, Germany; ^2^Department of Integrative and Complementary Medicine, Institute for Social Medicine, Epidemiology and Health Economics, Immanuel Hospital Berlin, Charité University Medical Center Berlin, 14109 Berlin, Germany

## Abstract

Acupuncture and meditation are promising treatment options for clinical pain. However, studies investigating the effects of these methods on experimental pain conditions are equivocal. Here, the effects of electroacupuncture (EA) and meditation on the submaximum effort tourniquet technique (SETT), a well-established, opiate-sensitive pain paradigm in experimental placebo research were studied. Ten experienced meditators (6 male subjects) and 13 nonmeditators (6 male subjects) were subjected to SETT (250 mmHG) on one baseline (SETT only) and two treatment days (additional EA contralaterally to the SETT, either at the leg on ST36 and LV3 or at the arm on LI4 and LI10 in randomized order). Numeric Rating Scale (NRS) ratings (scale 0–10) were recorded every 3 min. During baseline, meditation induced significantly greater pain tolerance in meditators when compared with the control group. Both the EA conditions significantly increased pain tolerance and reduced pain ratings in controls. Furthermore, EA diminished the group difference in pain sensitivity, indicating that meditators had no additional benefit from acupuncture. The data suggest that EA as a presumable bottom-up process may be as effective as meditation in controlling experimental SETT pain. However, no combined effect of both the techniques could be observed.

## 1. Introduction

Over the past years, naturopathic treatment strategies, such as acupuncture or meditation, have received increasing attention with regard to the treatment of various health conditions, especially pain [1–4]. However, there is still a substantial lack of established experimental pain models for the investigation of acupuncture or meditation that can be utilized in the laboratory. Moreover, experimental studies examining a possible interaction of both the techniques are currently not available although both the methods are often recommended and practiced simultaneously for the treatment of chronic pain syndromes in a multimodal integrative or complementary medicine setting. It can be speculated that a combination of these methods is most effective if they utilize, at least in part, different neurophysiological pathways to exhibit their analgesic effects. If the simultaneous application of meditation and acupuncture fails to show additive effects, then this could be interpreted as an evidence demonstrating that the same descending pain-modulating pathways are involved (e.g., at the level of the dorsal horn).

Meditation effects are often compared with other cognitive manipulations, such as hypnosis or expectancy. These interventions are known to influence the subjective experience of pain and the associated neuronal activity [5–8], particularly with regard to the emotional and functional aspects of pain. Overall, meditation states are comparatively well described neurobiologically [9–13]. Moreover, recent investigations examined the analgesic effect of meditation on experimental, laboratory paradigms, such as the cold pressure test [14, 15], thermal pain [11, 16], or noxious laser stimulation [10]. Nonetheless, clear evidence for a functional relationship between meditation practice and pain relief in experimental, laboratory pain models is still lacking. At the same time, experimental pain paradigms such as thermal pain tests (e.g., [17, 18]) or electrical stimulation [16, 19, 20] have been utilized in laboratory acupuncture research to investigate acupuncture or acupuncture-like TENS (transcutaneous nerve stimulation) analgesia. In the light of the German large-scale acupuncture trials, the results are equivocal [21–24].

In the presented study, the standard paradigm of placebo research, the submaximum effort tourniquet technique (SETT, [25]), was used as experimental pain stimulus to investigate both EA- and meditation-induced analgesia.

## 2. Methods

### 2.1. Subjects

The study was approved by the institutional review board of the Medical Institutions of the University of Duisburg-Essen, Germany (no. 07-3499). Twenty-three healthy young men (20–44 years) were recruited. Among them, 10 participants (6 males, mean age: 37.3 ± 5.3 years) who had a minimum of 2 years experience in Vipassana meditation after Goenka (practicing at least 3 h/week, mean meditation experience: 3304.6 ± 1893.6 h) were compared with the 13 control participants (6 males, mean age: 24.2 ± 3.9 years) without any meditation experience. All the subjects were naïve to both the experimental procedure and EA. Before participating in the study, all the subjects were screened for exclusion criteria, such as peripheral vascular abnormalities, hypo/hypertension, chronic pain syndromes, peripheral neuropathy, pregnancy, current medication, and alcohol/drug abuse. After explanation of the experimental procedure, each subject signed a standardized consent form. It was emphasized during the instructions that the participant could withdraw his or her study participation at any point without giving a reason. Each participant received an expense allowance.

### 2.2. Study Design

On their first visit to the laboratory, the study participants completed the experimental pain procedure (SETT) without any further treatment while on day 2 and 3, they received EA on the arm or leg in randomized order, in addition to SETT. Each examination was separated by at least 48 hours.

### 2.3. Pain Induction

**Figure 1:**
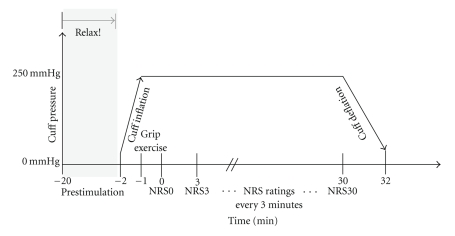
Schematic diagram of the pain induction procedure.

The SETT induces ischemic pain by inflating a blood pressure cuff on the arm for a prolonged period of time [25]. The SETT was performed according to the standard procedure in placebo research [26–28].  Figure 1 gives an overview of the design used. The subjects were asked to relax for 20 minutes by lying comfortably on an examination couch. Subsequently, they were asked to expose their nondominant arms above the bulk of the biceps/triceps. A standard blood pressure cuff was applied up to a point approximately 5 cm above the elbow crease, then the arm was elevated straight to the ceiling for 30 s, and afterwards, the cuff was rapidly inflated to 250 mmHg. The subjects were asked to lower their arms immediately after complete inflation and were instructed to perform 12 gripping exercises using maximal grip strength. The exercises were performed in a standardized manner by maintaining the grip for 1 s and relaxing for 1 s. The subjects were prompted by a standardized beep tone delivered by an mp3 player.

The study participants were prompted every 3 minutes to rate their pain on a numeric rating scale from 0 to 10 with 0 corresponding to “no pain” and 10 corresponding to “worst imaginable pain.” The first rating was given while bringing the arm back in the horizontal position. SETT time was limited to 30 minutes or a pain rating of 10. At the end of pain induction, the cuff was deflated slowly over a 2-minute period. On completion of the procedure, the cuff was carefully removed and the skin examined for any evidence of trauma. No evidence of such trauma or other side effects occurred throughout the study.

### 2.4. Acupuncture Procedure

**Figure 2:**
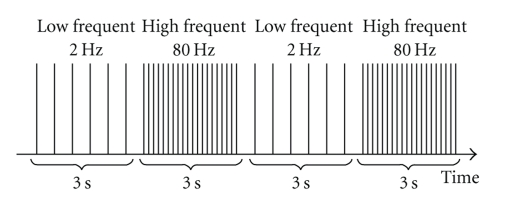
Schematic diagram of EA stimulation.

**Figure 3:**
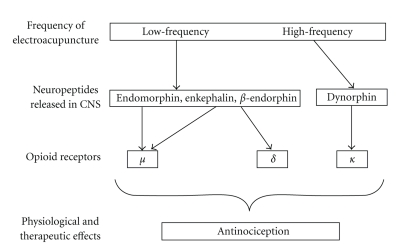
Schematic diagram displaying the opioid mechanisms of analgesia induced by EA (modified according to Han [29]).

Acupuncture was carried out by a physician licensed as an acupuncturist who was involved in neither data collection nor analysis. Common analgesic acupoints were selected for acupuncture treatment (leg condition: ST 36 and LV3, arm condition: LI4 and LI10). All acupoints were located contralaterally to the SETT. Before needle insertion, the acupoints' surroundings were pressed by the nonpuncturing index finger and thumb to exactly locate the acupoints. Afterwards, needling was performed with 0.25 × 25 mm stainless steel needles. All needles were inserted perpendicularly, with about 1-2 cm depth at LI4, LI10, and LV3, and about 2-3 cm depth at ST36. DeQi feeling was caused by rotating the needle clockwise and counterclockwise with a 180–360° amplitude for each rotation and for about 5–10 s of total stimulation. Stimulation was stopped when the subjects indicated that they achieved DeQi feeling. They were told before the treatment that DeQi is a dull, maybe hot or slightly sore sensation as a result of needle stimulation. Afterwards, the needles were connected to a standard EA device (cefar acus4). Stimulation was given 20 minutes prior to and throughout the SETT. EA was chosen because it is a rather strong acupuncture intervention, which can be applied steadily. Stimulation was conducted with low (2 Hz) and high (80 Hz) frequencies in alternating one-phase-square wave pulses (see Figure 2). The stimulation time of each pulse lasted for 180 *μ*s (pulse duration), and the duration of each phase was 3 s. According to Han [29], the analgesic effect of this mode of stimulation was found to be significantly more effective than pure low- or pure high-frequency stimulation (see Figure 3). Stimulation intensity (mA) was adjusted by asking the participants when the stimulation was perceived as strong and slightly painful but still endurable. In our study, the set stimulation intensities did not exceed the maximum of 2.1 mA.

### 2.5. Meditation

Different styles of meditation have proved to deliver “improvements in the functioning of mind and brain…consistent with those observed in mental health” [30]. A very old and strict form of meditation is the mainly concentrative Vipassana practice after the tradition of Goenka, which has its origin in Buddhism and is one of India's most ancient techniques of meditation [31]. Vipassana focuses on the connection between body and mind and is supposed to force a highly disciplined attention to the physical sensations that “instantly form the spiritual sensations.” Experienced Vipassana meditators were selected for the study because of the explicit distance to bodily and emotional experience in this meditation technique. All the meditators were asked to meditate throughout the whole experimental session on all three occasions.

### 2.6. Statistics

The results were analyzed using analysis of variance (ANOVA) for repeated measures with Greenhouse-Geisser correction when necessary. Post hoc comparisons were made by Bonferroni *α*-adjusted *t*-tests (two-sample *t*-tests for independent groups and matched-pairs *t*-tests where appropriate). Owing to multiple testing, adjusted significance level was set to *α* = 0.006.

## 3. Results

No subject withdrew from the study. Additionally, no side effects such as skin trauma owing to pain induction occurred during the study.

### 3.1. Numeric Rating Scale (NRS) Ratings

**Figure 4:**
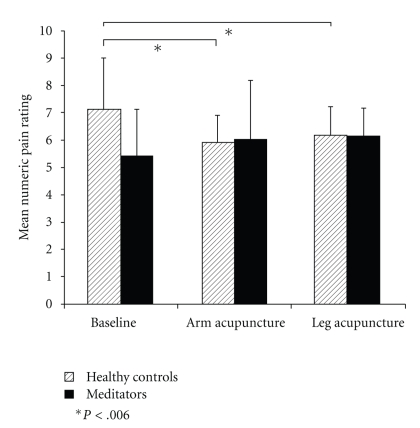
Mean numeric pain ratings with error bars indicating standard deviations, separated into the two groups on the three experimental occasions.

In case the participants gave a rating of “10” prior to the time limit of 30 minutes, numeric pain ratings interpolated to 10 for the remaining pain rating points. The average rating over time was calculated by dividing the cumulative rating by 11.  Figure 4 summarizes the averaged NRS ratings for the two groups at the three pain assessment days.

Greenhouse-Geisser corrected ANOVA showed a significant interaction between group and treatment condition with *F*(21,1) = 9.403, *P* = .002, and *ε* = 0.684. This interaction was mainly owing to treatment effects in the control group (Bonferroni adjusted two-sample *t*-test *α* = 0.006).  Table 1 summarizes the corresponding *P*-values.

**Figure 5:**
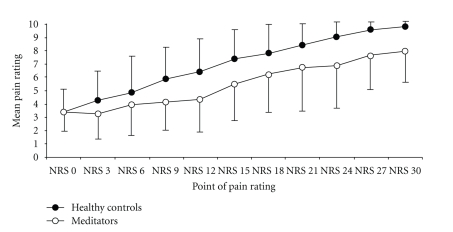
Baseline time course of mean numeric pain ratings with error bars indicating standard deviations, separated into the two groups.

A two-sample *t*-test for independent groups revealed a trend (see Figure 5 for the course of the pain rating during baseline session) but without any significant difference between the groups during baseline with *t*(21) = 1.99 and *P* = .059. There was no such trend for the other treatment conditions (arm condition: *t*(21) = −0.223 and *P* = .825; leg condition: *t*(21) = −0.068 and *P* = .946).

### 3.2. Break-Off Times

**Figure 6:**
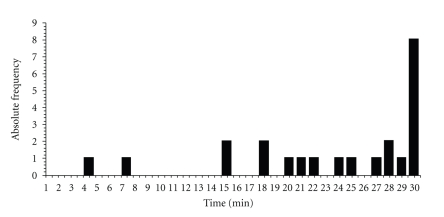
Absolute frequency of overall break-off times in the baseline condition.

Greenhouse-Geisser corrected ANOVA showed no significant interaction between the group and treatment condition with *F*(21,1) = 2.572, *P* = .112, and *ε* = .661, no main effect for treatment with *F*(21,1) = 0.762 and *P* = .425, or no main effect for the group with *F*(21,1) = 0.543 and *P* = .469. Therefore, there was no significant effect for the point of break-off. However, there was already a general and strong ceiling effect for the point at break-off at the baseline condition. A total of 56.5% of the participants (46.2% of these were controls and 53.8% were meditators) tolerated SETT pain for more than 25 min, and actually 34.8% (37.5% of these were controls and 62.5% were meditators) hit the time limit of 30 min (Figure 6). Therefore, a possible treatment or group effect was blurred by more than half of the data being zero for the differences between the baseline and treatment values.

### 3.3. Pain Tolerance

**Figure 7:**
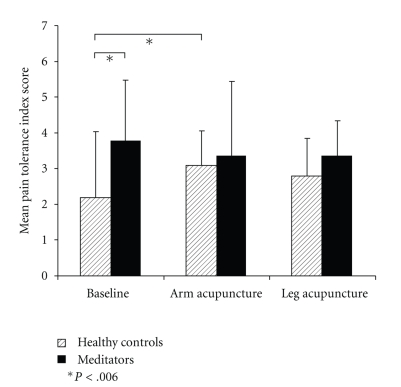
Mean pain tolerance index scores with error bars indicating standard deviations, separated into the two groups on the three experimental occasions.

Owing to the strong ceiling effect concerning the break-off times, pain tolerance index scores (defined as the point of break-off divided through the rating at that point) were calculated to derive a measure that takes rating and time into account. ANOVA revealed a significant interaction between the group factor and treatment condition with *F*(21,1) = 4.120 and *P* = .023.  Figure 7 shows the tolerance index scores.

Bonferroni *α*-adjusted (*α* = 0.006) two-sample *t*-test for sampled groups indicated that this interaction was owing to a significant treatment effect of acupuncture on the arm in controls. There was a trend towards pain relief caused by leg acupuncture though owing to the *α*-adjustment, it did not reach a significant level. Table 2 gives the corresponding *p* values.

A two-sample *t*-test for independent groups revealed a significant difference between the groups during baseline with *t*(21) = −2.596 and *P* = .032 but not during the other treatment days.

## 4. Discussion

**Figure 8:**
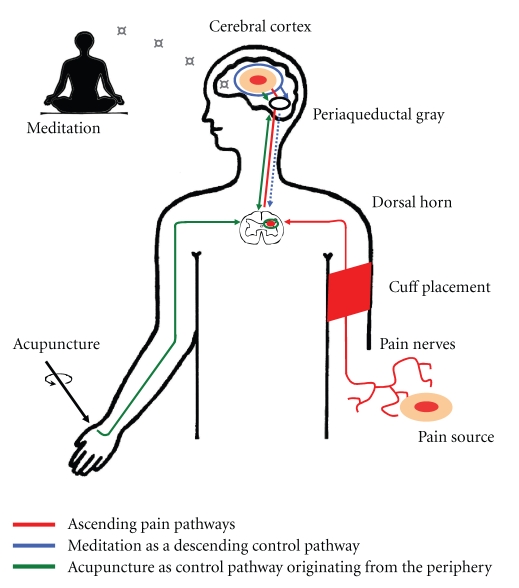
Simplified schematic diagram contrasting the mechanisms of acupuncture as a bottom-up and meditation as a top-down process.

Meditation as well as EA was shown to substantially control SETT-induced pain while no synergetic effect of both the techniques was observed (Figure 8). As EA was similarly effective at both the arm and leg, it is likely that supraspinal mechanisms, such as the placebo effect (e.g., [32, 33]) or the spinomedullary Diffuse Noxious Inhibitory Controls (DNICs, [34]) were involved.

SETT-induced pain was chosen because (i) the test is opiate-sensitive [26–28], and acupuncture analgesia was previously shown to be partially mediated through endogenous opiate-dependent pathways [35–42], (ii) it has been suggested that acupuncture analgesia may represent a placebo mechanism and the SETT presents a well-established paradigm out of placebo research, and (iii) acupuncture analgesia needs time to unfold its effects. Although there is a foremost evidence [43] demonstrating that analgesic acupuncture effects can also be observed in a threshold paradigm, we expected a more robust effect using a tolerance paradigm. Furthermore, pain tolerance is more similar to patients' painful experiences.

Clinical acupuncture effects have been discussed to be placebo effects, because “verum” and “sham” acupuncture were similarly effective in the German acupuncture studies (e.g., [21–24]). However, even though it was a standard procedure to utilize minimal or non-point-specific acupuncture as a control, there are now substantial doubts whether these conditions are really inactive for the treatment of pain [44–47]. Support for the notion that the German acupuncture trials differ from other trials on pain comes from a recently updated Cochrane review [48]. The authors were unable to reveal a placebo effect across a wide variety of conditions, including pain. The effect on pain was particularly variable, also among trials with low risk of bias. Among these, the four similarly designed German acupuncture trials reported the largest effects whereas three other pain trials reported low or no effect. This is a strong hint to the fact that the assumed sham control condition in these trials was active. In the light of a possible misinterpretation of “sham” acupuncture to be an inactive condition, former attempts to investigate acupuncture analgesia with the SETT, revealing no significant difference between true and sham acupuncture [49], have to be re-evaluated. In the study by Barlas et al., both the conditions showed a substantial reduction in pain ratings. Moreover, a study from our own group showed that EA was as effective as a single dose of an orally administered opiate in reducing SETT pain while there was no effect of a placebo pill or Ibuprofen [50]. In summary, we conclude that placebo effects in acupuncture trials are largely overestimated and that the acupuncture effects seen in our study are not owing to placebo effects.

Meditation-induced analgesia is in line with numerous findings showing that other cortically mediated or “top-down” mechanisms, such as expectation, emotion, or attention, affect the neuronal activity of brain regions involved in descending pain inhibition [5, 51–53]. People suffering from pain are active in trying multiple treatments, including self-care strategies [54], and often use them in combination.

The lack of synergetic effects of meditation and EA in this study is interesting and contradicts the theoretical assumption that hypnosis, another top-down process, and acupuncture are expected to reveal synergistic effects [8]. However, our findings are in line with the recent exciting data from the study by Eippert et al. showing that the cortically induced antinociceptive placebo response utilizes a spinal pathway [55]. “Top-down” mechanisms could therefore induce presynaptic inhibition at the level of the dorsal horn [56] and thus have a direct influence on the incoming sensory information.

It is possible to observe a physiological “ceiling” effect in our data owing to the fact that both the interventions utilize a final common pathway. Furthermore, the lack of a synergetic effect of EA and meditation in our study may even reflect a reciprocal inhibition. It has been shown in humans that expectation with regard to pain can change the intensity of spinal nociceptive responses. In particular, the expectation of hyperalgesia can completely block the normal analgesic response induced by DNIC [5]. As DNIC is a likely mechanism in the mode of action of acupuncture analgesia, an antianalgesic interaction at the level of the periaqueductal gray and the rostroventral medulla must also be considered. Nonetheless, this hypothesis still remains tentative and has to be investigated further.

The results presented here are based on a rather small sample, and there are some other limitations of the study. As a complex intervention, acupuncture treatment is almost impossible to blind. Therefore, as in almost all acupuncture trials, EA was blinded neither to participants nor to the acupuncturist. However, this holds true for the group of meditators as well, who did not show any benefit from acupuncture. Furthermore, factors other than the actual meditative state, for example, meditators' lifestyle, could have influenced the results. The fact that meditators exhibit permanent brain alterations in the brainstem [13] also and that DNIC is mediated through spinomedullary circuits [34], may have contributed to the group differences.

A further limitation of the study was the ceiling effect observed for the break-off times owing to a rather strict time limit. When compared with the ratings that are likely to represent the emotional aspects of pain processing, the break-off times correspond more to the behavioral aspects of pain, for example, two subjects giving the same pain rating might still have different break-off times. As there is no consensus on how to analyze SETT data if ceiling effects occur, we decided to calculate a pain tolerance index score that takes both the pain ratings as well as the break-off times into account. Thus, the sensational and behavioral aspects of pain processing were expressed in one index.

In conclusion, the standard pain paradigm of placebo research, the SETT, has been shown to be a valid tool in experimental acupuncture as well as meditation research. EA on the leg increased pain tolerance in healthy controls to 25.6% and EA on the arm increased pain tolerance to 38.4% while meditators reached a 69.7% higher pain tolerance when compared with the baseline condition of the healthy controls. These are substantial effects supporting the effectiveness of both the procedures in pain control. To our knowledge, this is the first investigation directly comparing the effect of acupuncture and meditation as two commonly recommended methods of therapeutic pain control in an experimental, laboratory pain procedure.

## Figures and Tables

**Table 1:** Test statistics for two-sample-*t*-test for sampled groups.

Group	Tested variables	Mean	SD	*t*-values	df	*P*-values
Controls	Mean rating base and mean rating with arm acupuncture	1.15035	.78211	5.303	12	.000*
	Mean rating base and mean rating with leg acupuncture	.88811	.79807	4.012	12	.002*
	Mean rating with arm and leg acupuncture	−.26224	.69163	−1.367	12	.197

Meditators	Mean rating base and mean rating with arm acupuncture	−.59091	1.4915	−1.298	9	.226
	Mean rating base and mean rating with leg acupuncture	−.72727	1.79403	−1.282	9	.232
	Mean rating with arm and leg acupuncture	−.13636	.58525	−.737	9	.480

**Table 2:** Test statistics for two-sample-*t*-test for sampled groups.

Group	Tested variables	Mean	SD	*t*-values	df	*P*-values
Controls	Pain tolerance base and pain tolerance with arm acupuncture	−.85154	.80883	−3.796	12	.003*
	Pain tolerance base and pain tolerance with leg acupuncture	−.56722	.79011	−2.588	12	.024
	Pain tolerance with arm and leg acupuncture	−.26224	.69163	−1.367	12	.197

Meditators	Pain tolerance base and pain tolerance with arm acupuncture	.44000	1.50569	.924	9	.380
	Pain tolerance base and pain tolerance with leg acupuncture	.40529	7.75281	.731	9	.483
	Pain tolerance with arm and leg acupuncture	−.13636	.58525	.131	9	.480
